# Formulation and Characterization of Poly (Ethylene Glycol)-Coated Core-Shell Methionine Magnetic Nanoparticles as a Carrier for Naproxen Delivery: Growth Inhibition of Cancer Cells

**DOI:** 10.3390/cancers14071797

**Published:** 2022-04-01

**Authors:** Faten Eshrati Yeganeh, Amir Eshrati Yeganeh, Mohammad Yousefi, Bahareh Farasati Far, Iman Akbarzadeh, Dmitry Olegovich Bokov, Kaamran Raahemifar, Madjid Soltani

**Affiliations:** 1 Department of Chemistry, Science and Research Branch, Islamic Azad University, Tehran 1477893855, Iran; ffyeganeh@gmail.com; 2 Department of Microbiology, Noor Dahesh Institute of Higher Education, 45789427600 Meymeh, Iran; amireshratiyegane@gmail.com; 3 Department of Chemistry, Faculty of Pharmaceutical Chemistry, Tehran Medical Sciences, Islamic Azad University, 899854961 Tehran, Iran; myousefi50@iausr.ac.ir; 4 Department of Chemistry, Iran University of Science and Technology, 1684613114 Tehran, Iran; bahar.ferasati@gmail.com; 5 Department of Chemical and Petrochemical Engineering, Sharif University of Technology, 1458889694 Tehran, Iran; imanakbarzadeh71@yahoo.com; 6 Institute of Pharmacy, Sechenov First Moscow State Medical University, 119991 Moscow, Russia; bokov_d_o@staff.sechenov.ru; 7 Laboratory of Food Chemistry, Federal Research Center of Nutrition, Biotechnology and Food Safety, 2/14 Ustyinsky pr., 109240 Moscow, Russia; 8 Faculty of Science, School of Optometry and Vision Science, University of Waterloo, Waterloo, ON N2L 3G1, Canada; 9 Data Science and Artificial Intelligence Program, College of Information Sciences and Technology (IST), Penn State University, State College, PA 16801, USA; 10 Department of Chemical Engineering, Faculty of Engineering, University of Waterloo, 200 University Ave W, Waterloo, ON N2L 3G1, Canada; 11 Center for Biotechnology and Bioengineering (CBB), University of Waterloo, Waterloo, ON N2L 3G1, Canada; 12 Department of Electrical and Computer Engineering, Faculty of Engineering, University of Waterloo, Waterloo, ON N2L 3G1, Canada

**Keywords:** drug delivery, Ni_1−x_Co_x_Fe_2_O_4_ NPs, methionine, PEGylating, MTT assay, cell line

## Abstract

**Simple Summary:**

Naproxen was loaded onto a magnetic nanoparticle coated with polyethylene glycol. Magnetic nanoparticles (MNPs) were used in this study to develop a smart naproxen delivery system. One of the most potent COX-1 and COX-2 inhibitors is naproxen, which belongs to the NSAID family of drugs. Although this drug has a short half-life, it has considerable toxicities and side effects on gastrointestinal tissues. The significant potential of our proposed nanocarrier for biomedical applications has been widely recognized; we modified MNPs to attach to this drug via disulfide bonds, promote the selective release of naproxen in inflammatory cells, and prevent adverse effects on the digestive system. It was found that the cytotoxicity of the drug was lowered by this change, which prevented unspecific protein binding.

**Abstract:**

An efficient and selective drug delivery vehicle for cancer cells can remarkably improve therapeutic approaches. In this study, we focused on the synthesis and characterization of magnetic Ni_1−x_Co_x_Fe_2_O_4_ nanoparticles (NPs) coated with two layers of methionine and polyethylene glycol to increase the loading capacity and lower toxicity to serve as an efficient drug carrier. Ni_1−x_Co_x_Fe_2_O_4_@Methionine@PEG NPs were synthesized by a reflux method then characterized by FTIR, XRD, FESEM, TEM, and VSM. Naproxen was used as a model drug and its loading and release in the vehicles were evaluated. The results for loading efficiency showed 1 mg of Ni_1−x_Co_x_Fe_2_O_4_@Methionine@PEG NPs could load 0.51 mg of the naproxen. Interestingly, Ni_1−x_Co_x_Fe_2_O_4_@Methionine@PEG showed a gradual release of the drug, achieving a time-release up to 5 days, and demonstrated that a pH 5 release of the drug was about 20% higher than Ni_1−x_Co_x_Fe_2_O_4_@Methionine NPs, which could enhance the intracellular drug release following endocytosis. At pH 7.4, the release of the drug was slower than Ni_1−x_Co_x_Fe_2_O_4_@Methionine NPs; demonstrating the potential to minimize the adverse effects of anticancer drugs on normal tissues. Moreover, naproxen loaded onto the Ni_1−x_Co_x_Fe_2_O_4_@Methionine@PEG NPs for breast cancer cell lines MDA-MB-231 and MCF-7 showed more significant cell death than the free drug, which was measured by an MTT assay. When comparing both cancer cells, we demonstrated that naproxen loaded onto the Ni_1−x_Co_x_Fe_2_O_4_@Methionine@PEG NPs exhibited greater cell death effects on the MCF-7 cells compared with the MDA-MB-231 cells. The results of the hemolysis test also showed good hemocompatibility. The results indicated that the prepared magnetic nanocarrier could be suitable for controlled anticancer drug delivery.

## 1. Introduction

Cancer includes a variety of diseases in which the growth of malignant cells has the potential to spread to other parts of the body [1,2,3]. One of the most common cancers is breast cancer in women; most deaths from breast cancer are due to its drug resistance and the potential for metastasis to distant organs [4,5,6]. There are several pathways involved in the modulation of breast cancer. One of these pathways is the use of nanoparticles (NPs) carrying anticancer agents that can be delivered to the targeted tumor and the treatment of breast cancer [7,8].

Drug delivery is a major topic of research in biomedicine that controls drug delivery and therapeutic drugs to target cells [9,10,11]. However, nanotechnology creates products that exhibit novel properties at nanoscale levels, which offer new tools for biologists [12,13,14]. Recently, the use of various organic–inorganic hybrid NPs because of advances in nanotechnology has drawn significant attention [15]. Studies have shown that modified magnetic NPs can be used in vivo as they increase the efficacy of drug delivery [16].

Magnetic NPs (MNPs), due to their potential in medical applications, have received extensive attention in biomedical communities [17,18,19]. They have a spinel structure with unique properties such as a considerable specific surface area, a small size, high saturation magnetization, and controlled magnetic behavior [20,21,22,23]. Amongst the magnetic NPs (MNP), cobalt ferrite (CoFe_2_O_4_) has drawn significant attention because the change of metals in its composition can substantially affect its physical and chemical characteristics as well as its biocompatibility to serve as a drug delivery system [22,24,25]. Spinel ferrites (SFs) can be used in medical diagnostics and therapy such as magnetic resonance imaging, controlled drug delivery, gene delivery, bioseparation, and therapeutic hyperthermia [26,27].

Biomedical applications must modify the surface of magnetic NPs to make them more stable before they are used for drug delivery to improve the limitations of these NPs, which include their destabilization effect and non-specific uptake by the RES; the size of magnetic NPs is also limited by the main immunogenic region [28,29,30,31]. Surface modification is required for biomedical applications to begin coating them with stimuli-responsive magnetic NPs that respond when exposed to external stimuli and can generate physicochemical changes in the structure that favor controlled drug release or release at a specific place [32,33,34]. Recently, the use of various organic–inorganic hybrid nanoparticles because of advances in nanotechnology has drawn significant attention. Methionine is one of the most crucial and primary biocompatible amino acids in the human body and it has been specialized for physiological purposes in vivo. Methionine has two active functional groups (-COOH and -NH_2_) that can be used for metal atom conjugation [35,36,37]. The conjugation of amino acids with polyethylene glycol (PEG) shows important therapeutic benefits. The use of NPs with PEG coatings inside biological samples has several advantages. For in vivo applications, PEG coatings can increase the particle circulation time by reducing protein and cell adsorption on the particles and decreasing the clearance rate of “PEGylated” materials.

Herein, we report the magnetic nanoformulations in which Ni_1−x_Co_x_Fe_2_O_4_ NPs were coated with methionine as an inner layer and polyethylene glycol as an outer layer. NSAIDs (non-steroidal anti-inflammatory drugs) such as naproxen, which are potent COX-1 and COX-2 inhibitors [38], have been studied; drug loading and release behavior using naproxen as a drug model on Ni_1−x_Co_x_Fe_2_O_4_@Methionine@PEG NPs have not been previously studied. MTT assays with different doses and incubation times were used to determine cytotoxicity in vitro using this as a carrier.

## 2. Materials and Methods

### 2.1. Materials

To prepare the Ni_1−x_Co_x_Fe_2_O_4_@Methionine@PEG NPs, FeCl_3_·6H_2_O (CAS #: 10025-77-1), CoCl_2_·6H_2_O (CAS #: 7791-13-1), NiCl_2_·6H_2_O (CAS #:7791-20-0), NaOH (CAS #: 1310-73-2), and methionine (CAS #: 63-68-3) were used as an inner shell of magnetic NPs; polyethylene glycol 6000 (PEG-6000) (CAS #: 25322-68-3) was used as an outer layer. Penicillin-streptomycin (CAS #: P4333), thiazolyl tetrazolium (CAS #: 298-93-1), fetal bovine serum (FBS) (CAS #: 12103C), and dimethyl sulfoxide (CAS #: 67-68-5) were purchased from Merck, Germany. MCF-7, MCF10A, and MDA-MB-231 cell lines were obtained from the Pasteur Cell Bank (Tehran, Iran). Naproxen was purchased from Daroo-Pakhsh Co. (Tehran, Iran). All the chemicals were obtained from Merck (Berne, Germany), and all were used as received without any purification.

### 2.2. Preparation of Methionine-Coated Magnetite (Ni_1−x_Co_x_Fe_2_O_4_@Methionine)

In a typical experiment to synthesize an Ni_1−x_Co_x_Fe_2_O_4_ NP coated with methionine using the reflux method, briefly 1.621 g FeCl_3_, 0.713 g CoCl_2_, and 0.713 g NiCl_2_ were stirred for 15 min in 90 mL of deionized water under an N_2_ atmosphere. The mixture was stirred followed by the addition of 25 mL of NaOH (1.5 M) until the mixture reached a pH of around 12. When the precipitate was obtained, magnetic stirring was used to dissolve 0.5 g of methionine in deionized water. A 70–80 °C heat was applied to the mixture and it was allowed to reflux for two hours. Magnetic separation was used to collect the final products and they were rinsed several times with a solution of ethanol and deionized water. The same experimental technique was used to produce bare Ni_1−x_Co_x_Fe_2_O_4_ NPs without methionine.

### 2.3. Preparation of PEG-Coated Magnetite (Ni_1−x_Co_x_Fe_2_O_4_@Methionine) NPs

A polyethylene glycol solution with a suitable concentration was prepared by dissolving PEG-6000 powder in a phosphate-buffered saline (PBS) solution. The mixture of the PBS solution and magnetic (Ni_1−x_Co_x_Fe_2_O_4_@Methionine) NPs was vigorously stirred for 30 min and then centrifuged at 6000 rpm to obtain the final NPs [29].

### 2.4. Characterization

An X-ray diffraction (XRD) analysis of the samples was recorded by an A X’ Pert Pro MPD, Panalytical (Amsterdam, Netherlands) with Cu Kα radiation (λ = 1.54060 Å) and with a 2θ range of 10–80° at room temperature. The emissions from the field of the NPs were sized and morphologically characterized using scanning electron microscopy (FESEM) and a transmission electron microscope (Zeiss EM900 Transmission Electron Microscope, Cologne, Germany). It was possible to obtain infrared spectra in the 4000–400 cm^−1^ range by using a BRUKER spectrometer (VERTEX 70). As a function of the soaking time, the amount of adsorbed and released medication was measured with a UV-Vis spectrophotometer (Shimadzu, UV-1700 Pharma spec, Tokyo, Japan). A Quantum Design MPMS-XL-7 superconducting quantum interference device (SQUID) with an external magnetic field assessed the magnetic characteristics of the NPs produced at an ambient temperature.

### 2.5. In Vitro Loading and Release of Naproxen

First, 0.1 g of nanoparticles (Ni_1−x_Co_x_Fe_2_O_4_@Methionine, Ni_1−x_Co_x_Fe_2_O_4_@Methionine@PEG) were dispersed in a PBS solution. We then prepared different contents of naproxen with 20 mL of a buffer of pH 7.4. The naproxen solution was added to the initial solution and the mixture stood for 24 h whilst being constantly agitated. After that, the dispersion was centrifuged at 6000 rpm and washed twice to collect the naproxen-loaded NPs; the supernatant was saved for estimating the drug loading concentration. UV-Vis spectroscopy was used to determine the loading capacity at a wavelength of 230 nm to assess the remaining drugs in the supernatants. The loading capacity was then computed using a standard calibration curve with various known drug concentrations. Equation (1) was used to compute the amount of loaded naproxen:

(1) $$\mathrm{Loading}\;(\%)=(C_{0}V_{0}-C_{t}V_{t}\alpha )/(w)\times 100$$

where *C*_0_ indicates the initial concentration of naproxen, *C_t_* represents the drug concentration determined by the standard naproxen curve, *α* is the dilution ratio, *V*_0_ and *V_t_* are the volumes of the aqueous phase in mL, and *w* (mg) is the weight of the nanocarrier.

Samples containing 20 mg of naproxen were placed in 20 mL of PBS at various pH values (5 and 7.4) and shaken at a constant rate (100 r/min) for a specific time in the dark at a constant temperature (37.5 °C). Each time the supernatant was removed, the volume of the new PBS with the same pH value was equally balanced. According to Equation (2), the UV-Vis method at a wavelength of 230 nm was used to calculate the proportion of naproxen that had been released:

(2) Drug release (%)=(Ce×V)/W×100

where *V* (mL) is the volume of buffer solution, *W* (mg) is the amount of drug loading, and *Ce* (mg/mL) is the concentration of naproxen in the supernatant.

We used various kinetic models such as zero-order, first-order, Higuchi, Korsmeyer–Peppas, and Hixson–Crowell.

### 2.6. In Vitro Cytotoxicity

On cancer cell lines MCF-7 and MDA-MB-231 as well as a normal cell line (MCF10A), the samples were tested for their cytotoxicity using an MTT assay, which is a commonly used technique. There were 2 × 10^4^ cells per well and 37 °C humidified incubators in 96-well plates with FBS and penicillin-streptomycin to seed the cells. After a 24 h incubation period, suspensions of various samples (0–35 µg/mL) were added to the medium and incubated for another 24 h period as well as for 48 and 72 h. In the next step, the contents of the 96-well plates were replaced by the MTT solution in each well after another 4 h of incubation at 37 °C in a 5% CO_2_ environment. To dissolve the purple formazan crystals, the medium was replaced and added to each well with 0.05 mL of dimethyl sulfoxide (DMSO) [39,40]. Finally, the absorbance of each well was measured using a microplate reader at 570 nm. The IC_50_ concentration and the cytotoxicity rate were estimated using Equation (3):

(3) Cell Survival rate=(absorbance of treated cells)/(absorbanece of control cells)×100.

### 2.7. Hemocompatibility Test

Fresh blood samples were taken from a healthy volunteer and centrifuged for 5 min at 2000 rpm to isolate the RBC cells. A total of 400 μL of the washed RBCs was diluted with 3.6 mL of PBS to produce the clear supernatant and then the cells were washed three times with PBS. Various concentrations (2000, 1750, 1500, 1250, 1000, and 750 μg·mL^−1^) of the Ni_1−x_Co_x_Fe_2_O_4_@Methionine@PEG nanoformulation in PBS (0.8 mL) was mixed with the diluted RBC suspension (0.2 mL) and kept without shaking for 2 h at room temperature. The negative control was PBS. Centrifugation and UV-Vis spectroscopy were used to measure the absorbance of the supernatant at 540 nm after 2 h to determine the percentage of hemolysis in the solution of each sample [41]. The following equation (Equation (4)) was used to estimate the percentage of hemolysis in each sample:

(4) Percent Hemolysis(%):(sample absorbance−negative control absorbance/positive control absorbance−negative control absorbance)×10.

## 3. Results

### 3.1. XRD Analysis

**Figure 1:**
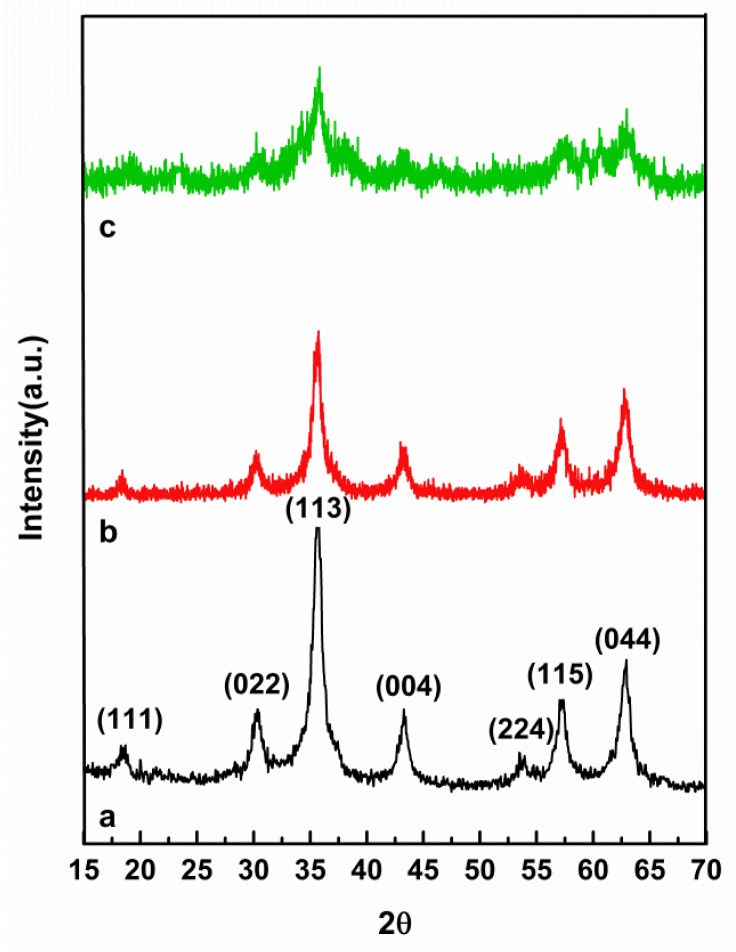
XRD patterns for the as-prepared magnetite Ni_1−x_Co_x_Fe_2_O_4_ NPs (**a**), methionine-coated Ni_1−x_Co_x_Fe_2_O_4_ NPs (**b**), and Ni_1−x_Co_x_Fe_2_O_4_@Methionine@PEG NPs (**c**).

The XRD patterns of the as-prepared magnetite Ni_1−x_Co_x_Fe_2_O_4_ NPs, methionine-coated samples, and PEG-coated magnetite (Ni_1−x_Co_x_Fe_2_O_4_@Methionine) NPs are shown in Figure 1, which confirmed that the Ni_1−x_Co_x_Fe_2_O_4_ nanoparticle peaked at 2θ = 18.72°, 30.35°, 35.70°, 43.33°, 53.71°, 57.23°, and 62.84° and could be indexed to the planes of (111), (022), (113), (004), (224), (115), and (044), respectively, which was in agreement with the theoretical values (JCPDS standard data, Card No. 98-001-6669). Furthermore, in the methionine-coated Ni_1−x_Co_x_Fe_2_O_4_ nanoparticle, the peaks were at 2θ = 18.39°, 30.24°, 35.69°, 43.17°, 53.64°, 57.23°, and 62.84 °, decreasing the 2theta values and indicating the entry of methionine into the network cavities and increasing the network space. Characteristic diffraction peaks corresponded with the crystal planes (111), (022), (113), (004), (224), (115), and (044), respectively (JCPDS standard data, card No. 98-001-6669) [29,37]. After the PEGylation of the core-shell of the Ni_1−x_Co_x_Fe_2_O_4_@Methionine NP with decreasing 2theta values, polyethylene glycol entered into the network cavities and the network space increased. However, the peaks were broad after capping with PEG.

### 3.2. FTIR Analysis

**Figure 2:**
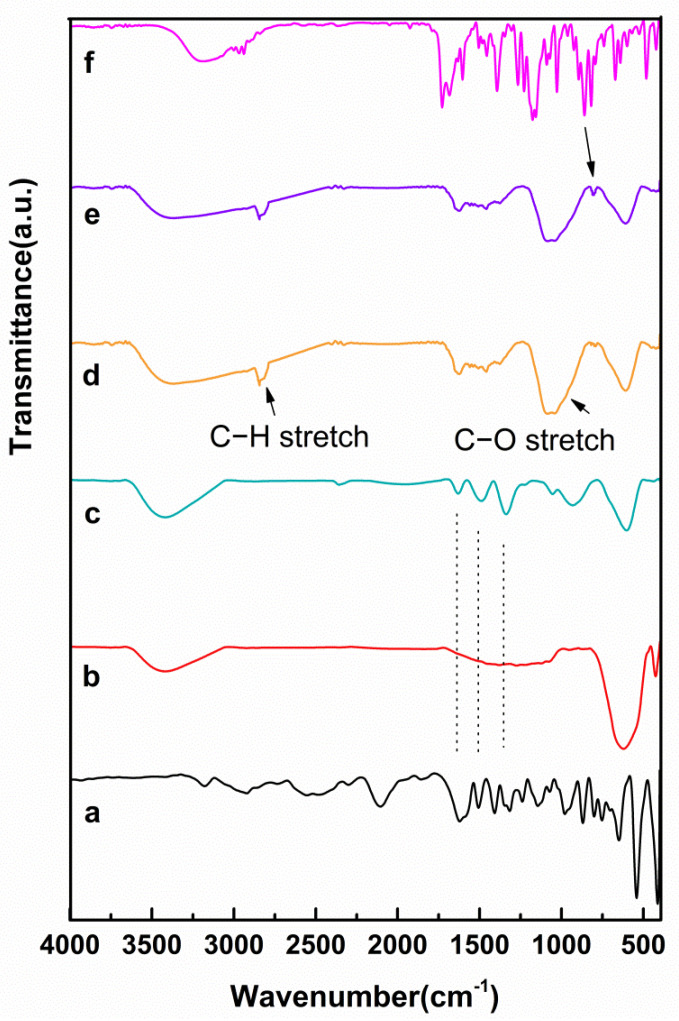
The FTIR spectra of (**a**) methionine, (**b**) Ni_1−x_Co_x_Fe_2_O_4_ NPs, (**c**) Ni_1−x_Co_x_Fe_2_O_4_@Methionine, (**d**) Ni_1−x_Co_x_Fe_2_O_4_@Methionine@PEG, (**e**) naproxen loaded onto the Ni_1−x_Co_x_Fe_2_O_4_@Methionine@PEG, and (**f**) pure naproxen.

The FTIR spectra of all samples were recorded using an FTIR spectrophotometer in the range of 400 to 4000 cm^−1^. As shown in Figure 2, methionine showed peaks at 1517 cm^−1^ (symmetric N–H bending), 1630 cm^−1^ (asymmetric N–H bending), 1419 cm^−1^, 1600 cm^−1^ (symmetric and asymmetrical stretching of COO), and 1232–1330 cm^−1^ (C–O band). The peaks of the tetrahedral and octahedral metal-oxygen complexes of Ni_1−x_Co_x_Fe_2_O_4_, which are mainly dependent on Fe–O distances, were located at 400 and 600 cm^−1^, respectively.

The absorption bands in the pure naproxen spectrum around 1028 cm^−1^ and 1264 cm^−1^ were due to symmetric Aryl–O stretching and asymmetric Aryl–O stretching, respectively. The spectra around 1090 cm^−1^ were related to C–O stretching. The peak in the region of 1481–1604 cm^−1^ was caused by aromatic C=C stretching and the peaks of 1682 cm^−1^ and 1728 cm^−1^ referred to the C=O H bonds and non-H bonds stretching, respectively.

In the curve, the O–H stretch band was approximately presented at 3404 cm^−1^, the C–H stretch band at 2835 cm^−1^, and the C–O stretch band at 1058 cm^−1^. These were are all from the PEG-coated Ni_1−x_Co_x_Fe_2_O_4_@Methionine NP, demonstrating the existence of a residual PEG in the final sample. As seen in Figure 2 in curve e, hydrogen bonds were thought to be present between naproxen and the analyzed NPs. Given the C-H stretching groups in polyethylene glycol and the presence of oxygen in the naproxen, this possibility was reasonable.

### 3.3. Magnetic Properties

**Figure 3:**
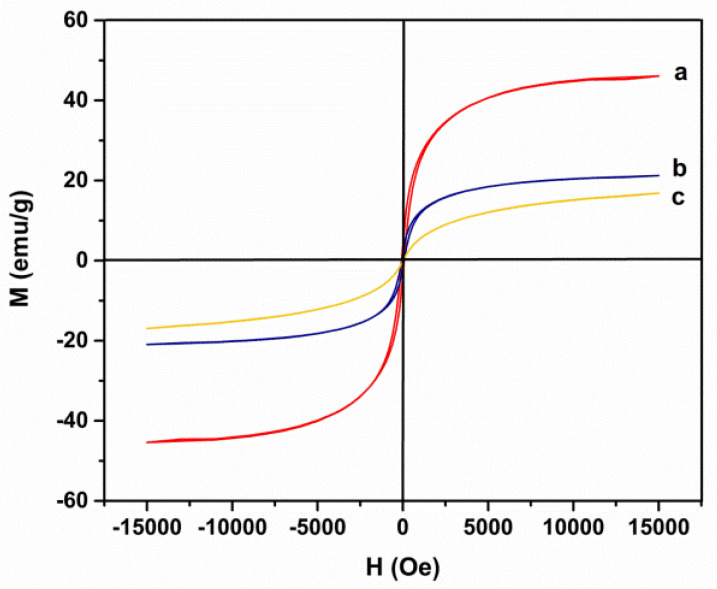
Magnetization curves for the as-synthesized (**a**) Ni_1−x_Co_x_Fe_2_O_4_ NPs, (**b**) Ni_1−x_Co_x_Fe_2_O_4_@Methionine NPs, and (**c**) Ni_1−x_Co_x_Fe_2_O_4_@Methionine@PEG NPs.

As can be seen in Figure 3, the magnetic properties of the synthesized Ni_1−x_Co_x_Fe_2_O_4_@Methionine NPs, Ni_1−x_Co_x_Fe_2_O_4_@Methionine@PEG NPs, and bare Ni_1−x_Co_x_Fe_2_O_4_ NPs were measured at room temperature, indicating that the Ni_1−x_Co_x_Fe_2_O_4_ NPs exhibited a superparamagnetic behavior. Figure 3a shows that the saturation magnetization of the bare Ni_1−x_Co_x_Fe_2_O_4_ NPs was estimated to be 46.09 emu/g. Similar behavior for the Ni_1−x_Co_x_Fe_2_O_4_@Methionine NPs was observed but with less magnetization of 21.86 emu/g (Figure 3b), and even less saturation magnetization for the Ni_1−x_Co_x_Fe_2_O_4_@Methionine@PEG NPs, which was estimated to be 16.26 emu/g.

### 3.4. Morphology Study

**Figure 4:**
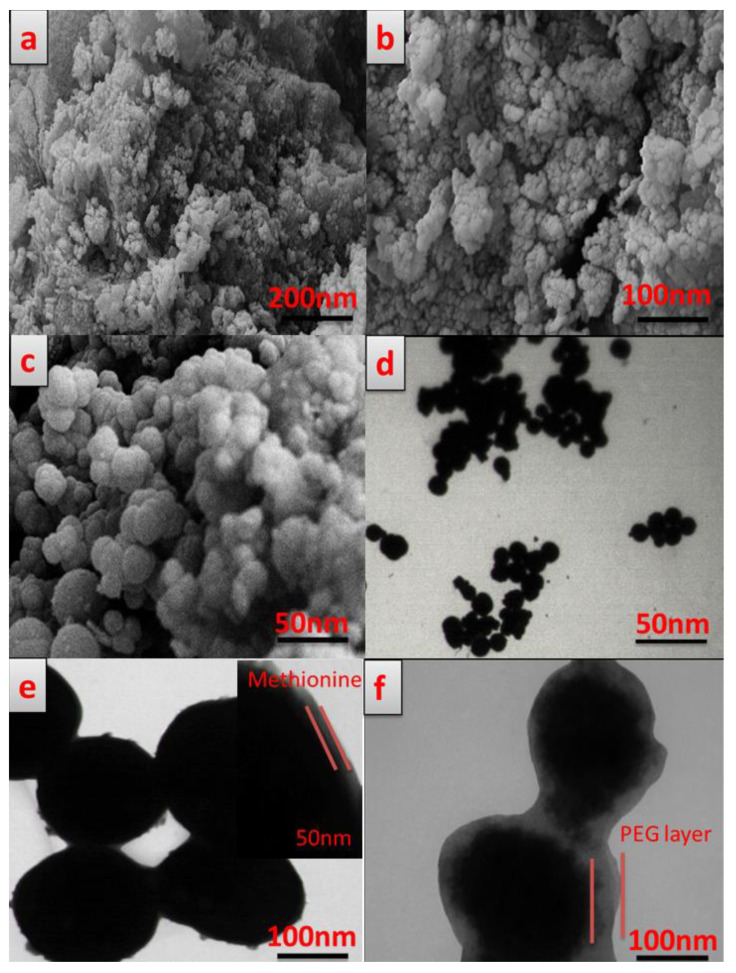
FESEM micrographs of Ni_1−x_Co_x_Fe_2_O_4_ NP (**a**), Ni_1−x_Co_x_Fe_2_O_4_@Methionine NP (**b**), and Ni_1−x_Co_x_Fe_2_O_4_@Methionine@PEG NP (**c**), and TEM images of Ni_1−x_Co_x_Fe_2_O_4_ NP (**d**), Ni_1−x_Co_x_Fe_2_O_4_@Methionine NP (**e**), and Ni_1−x_Co_x_Fe_2_O_4_@Methionine@PEG NP (**f**).

The SEM images of the synthesized Ni_1−x_Co_x_Fe_2_O_4_, Ni_1−x_Co_x_Fe_2_O_4_@Methionine, and Ni_1−x_Co_x_Fe_2_O_4_@Methionine@PEG NPs are shown in Figure 4a–c, which demonstrated that the magnetite nanoparticles were an irregular shape with an average particle size of about 13.3 nm. The Ni_1−x_Co_x_Fe_2_O_4_@Methionine and Ni_1−x_Co_x_Fe_2_O_4_@Methionine@PEG NPs were spherical and almost uniform in size with an average particle size of about 17–18 nm and 28–29 nm, respectively. Therefore, the average size of the nanoparticles made them suitable for drug delivery applications. Figure 4d–f shows the TEM images of the Ni_1−x_Co_x_Fe_2_O_4_, Ni_1−x_Co_x_Fe_2_O_4_@Methionine, and Ni_1−x_Co_x_Fe_2_O_4_@Methionine@PEG NPs, respectively. As shown in Figure 4d, the Ni_1−x_Co_x_Fe_2_O_4_ magnetic NPs had a slight agglomeration due to strong magnetic interactions between the NPs [29]. After coating the magnetic NPs with a layer of methionine as an inner layer, a light gray shell around the Ni_1−x_Co_x_Fe_2_O_4_ NPs could be seen and, in the next step, the thickness of the polymer shell of the nanoparticles could clearly be seen, which was increased by adding a polyethylene glycol solution as an outer layer.

### 3.5. Drug Loading and Release Behavior In Vitro

**Figure 5:**
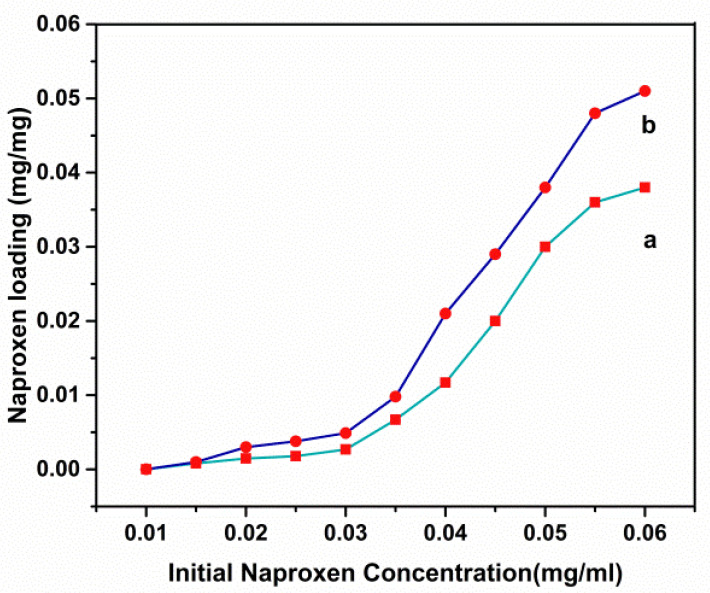
Loading capacity curves of naproxen on the Ni_1−x_Co_x_Fe_2_O_4_@Methionine NPs (**a**) and Ni_1−x_Co_x_Fe_2_O_4_@Methionine@PEG NPs (**b**) at different initial concentrations of the drug.

Figure 5 shows the amount of naproxen that could be absorbed into the body. Using these findings, it was concluded that the maximum amount of naproxen adsorption of the Ni_1−x_Co_x_Fe_2_O_4_@Methionine nanoparticles was 0.038 mg/mg when the initial naproxen concentration was 0.06 mg/mL and the maximum amount of naproxen adsorption of the Ni_1−x_Co_x_Fe_2_O_4_@Methionine@PEG nanoparticles was 0.051 mg/mg. As a result, 1 mg of Ni_1−x_Co_x_Fe_2_O_4_@Methionine NPs loaded 0.38 mg and 1 mg of Ni_1−x_Co_x_Fe_2_O_4_@Methionine@PEG NPs loaded 0.51 mg of the drug, respectively.

**Figure 6:**
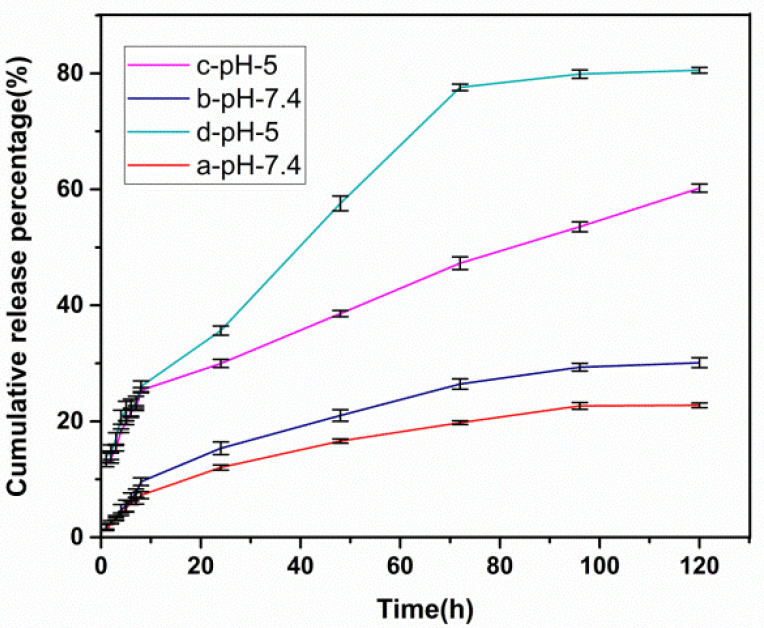
Cumulative release curves of naproxen from naproxen-loaded Ni_1−x_Co_x_Fe_2_O_4_@Methionine NPs at pH 5 (**c**) and pH 7.4 (**b**); Ni_1−x_Co_x_Fe_2_O_4_@Methionine@PEG NPs at pH 5 (**d**) and pH 7.4 (**a**). Data are expressed as mean ± SD (*n* = 5).

Figure 6 represents the percentage of naproxen released from the Ni_1−x_Co_x_Fe_2_O_4_@Methionine and Ni_1−x_Co_x_Fe_2_O_4_@Methionine@PEG NPs synthesized as described above (37 °C). For the simulated tumor environment, pH values of −5 and −7.4 were specified. In 120 h, the release rates of both carriers were lower in neutral solution settings (pH 7.4) than in acid solution conditions (pH 5). It was also found that, under the same conditions (pH 5), the drug was released about 20% faster in the Ni_1−x_Co_x_Fe_2_O_4_@Methionine@PEG NPs than in the Ni_1−x_Co_x_Fe_2_O_4_@Methionine NPs. The zero-order, first-order, Higuchi, and Korsmeyer–Peppas models were used in this investigation to systematically determine the release behavior of the naproxen-loaded NPs at different pH values (5 and 7.4). A higher linear regression coefficient (closer to 1) suggested the ideal sample release kinetic model, as shown in Table 1. The Higuchi model was used to calculate the release data for all samples.

### 3.6. Cytotoxicity Studies

**Figure 7:**
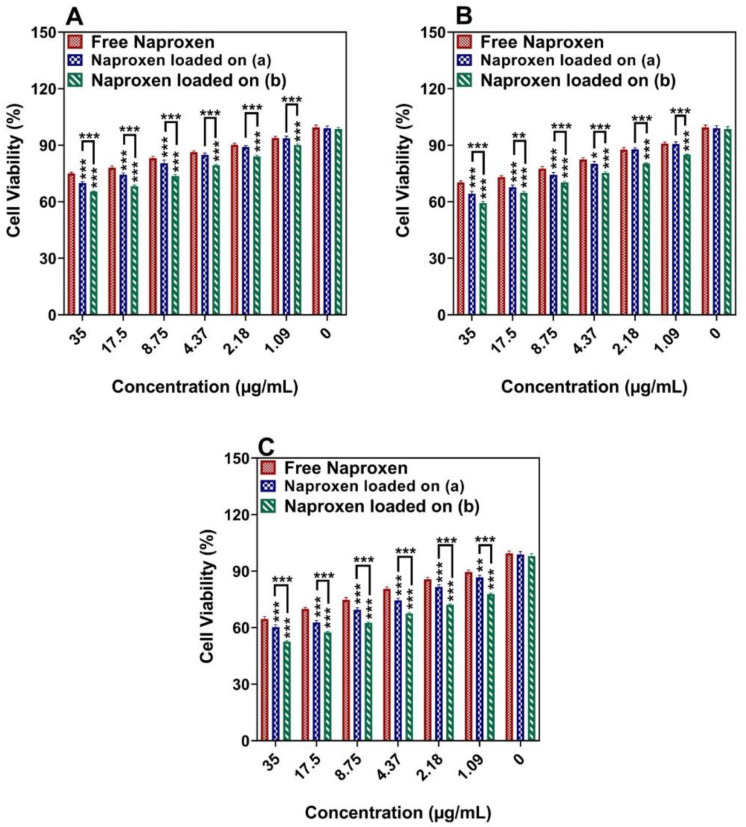
The effect of free drug and drug-loaded carriers (a) Ni_1−x_Co_x_Fe_2_O_4_@Methionine NPs and (b) Ni_1−x_Co_x_Fe_2_O_4_@Methionine@PEG NPs on the viability of MCF-7 cells after incubation for (**A**) 24 h, (**B**) 48 h, and (**C**) 72 h. Data are expressed as mean ± SD (*n* = 5) (*** *p* < 0.001, ** *p* < 0.01, * *p* < 0.05).

We chose human breast cancer cells (MCF-7 and MDA-MB-231) and normal cells (MCF10A) as cell models and tested the cytotoxicity after incubation three times for 24, 48, and 72 h with naproxen loaded onto Ni_1−x_Co_x_Fe_2_O_4_@Methionine NPs, free naproxen, and naproxen-loaded Ni_1−x_Co_x_Fe_2_O_4_@Methionine@PEG NPs, respectively. The MTT assay of the MCF-7 cells (Figure 7) showed naproxen loaded onto Ni_1−x_Co_x_Fe_2_O_4_@Methionine@PEG NPs had significant cell death, which was much higher than the naproxen loaded onto Ni1-xCoxFe_2_O_4_@Methionine; both carriers caused more cell death than free naproxen.

**Figure 8:**
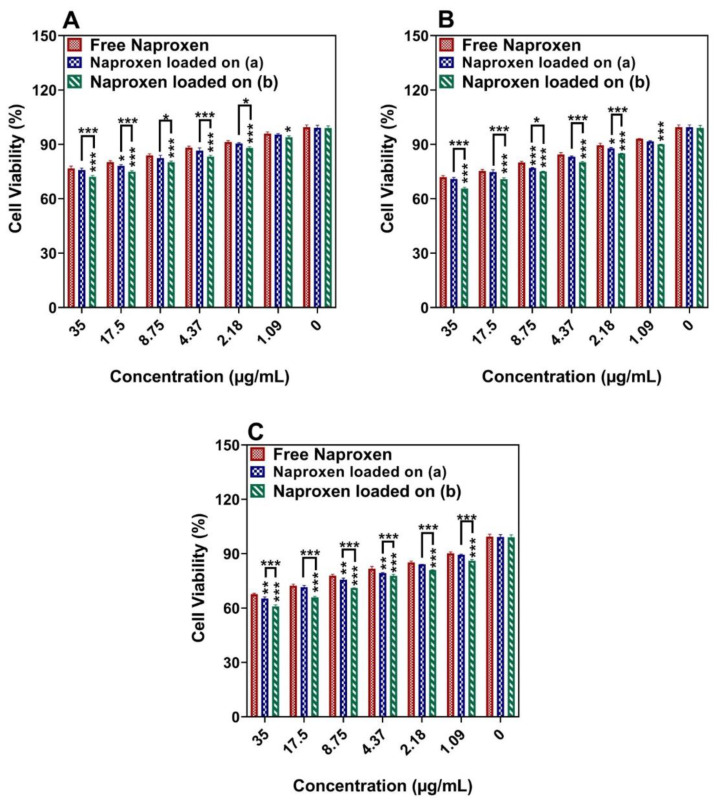
The effect of free drug and drug-loaded carriers (a) Ni_1−x_Co_x_Fe_2_O_4_@Methionine NPs and (b) Ni_1−x_Co_x_Fe_2_O_4_@Methionine@PEG NPs on the viability of MDA-MB-231 cells after incubation for (**A**) 24 h, (**B**) 48 h, and (**C**) 72 h. Data are expressed as mean ± SD (*n* = 5) (*** *p* < 0.001, ** *p* < 0.01, * *p* < 0.05).

**Figure 9:**
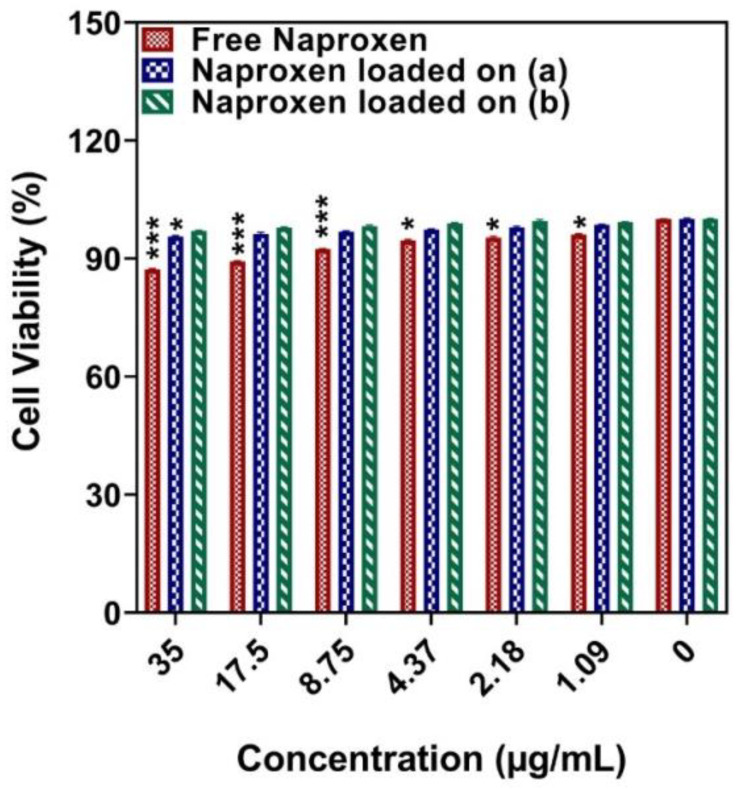
The effect of free drug and drug-loaded carriers (a) Ni_1−x_Co_x_Fe_2_O_4_@Methionine NPs and (b) Ni_1−x_Co_x_Fe_2_O_4_@Methionine@PEG NPs on the viability of normal MCF10A cells after incubation for 72 h. Data are expressed as mean ± SD (*n* = 5) (*** *p* < 0.001, * *p* < 0.05).

According to Figure 8, the MTT experiment of the MDA-MB-231 cells showed that naproxen loaded onto Ni_1−x_Co_x_Fe_2_O_4_@Methionine@PEG NPs killed cells faster than Ni_1−x_Co_x_Fe_2_O_4_@Methionine NPs and faster than the free drug (Figure 8 for 24, 48, and 72 h, respectively). According to the comparison of the two types of cancer cells, both carriers caused higher cell death in MCF-7 than in MDA-MB-231. As shown in Figure 9, the drug-loaded NPs did not cause any substantial toxicity in the normal MCF10A cells following a 72 h treatment, indicating that they were biocompatible enough to be used in drug delivery. When naproxen was loaded onto the carriers, the growth inhibition effect on the cancer cells improved their therapeutic possibilities. Table 2 summarizes the IC_50_ values of the substances against the MCF-7 and MDA-MB-231 cells, respectively.

### 3.7. Hemocompatibility Test

The biocompatibility of materials intended for use in the biomedical field must be appropriately evaluated. The hemolytic activity test is a reliable and scientific method for determining the biocompatibility of a synthetic material with living systems [42]. 

**Figure 10:**
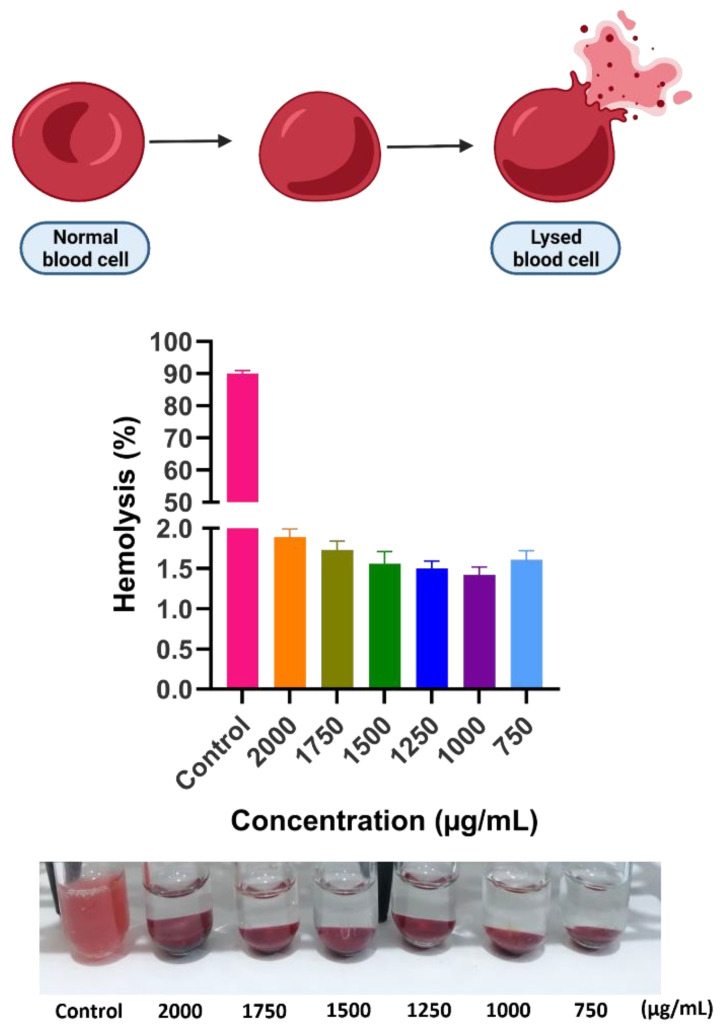
Hemolysis percentage of Ni_1−x_Co_x_Fe_2_O_4_@Methionine@PEG NPs with a positive control at different concentrations.

Nanoparticle hemocompatibility is undervalued despite the fact that most nanoparticles will meet blood at some point during their path through the body. Nanoparticles can affect the morphology of red blood cells (RBCs) or erythrocytes, resulting in hemolysis. Hemolysis happens when the cell membrane is ruptured and the cells are lysed. By inciting the immune system to suppress them, these negative interactions between nanoparticles and the bloodstream may increase inflammatory and autoimmune illnesses as well as infection and malignancy [43]. The blood compatibility of Ni_1−x_Co_x_Fe_2_O_4_@Methionine@PEG was examined in this study (Figure 10). Hemolysis percentages lower than 5% were recorded for all concentrations of Ni_1−x_Co_x_Fe_2_O_4_@Methionine@PEG (150, 125, 100, 75, 50, and 25 μg·mL^−1^), which are regarded as safe [44]. Nanoformulation has been demonstrated to be a safe method for the hemolysis of human erythrocytes. This may be due to the inclusion of PEG, which facilitates NP escape from the reticuloendothelial system and prevents macrophage scavenging. This is a solution to the unfavorable hemocompatibility of CoFe_2_O_4_, which has been shown in previous studies to have a hemolytic effect on human erythrocytes [45].

## 4. Discussion

In our previous experiments to produce magnetic Ni_1−x_Co_x_Fe_2_O_4_@Methionine nanoparticles, we occasionally found aggregation among the particles that may have been due to (interparticle) hydrogen bonding between the magnetic nanoparticles and methionine. The answer to the problems posed by the aggregations among the particles in an aqueous suspension is Steric hindrances; therefore, using PEG polymers (steric stabilizers) adsorbed or grafted onto the particle surfaces, contacts among the particles were prevented by the polymer chains extending into the medium. However, with the PEGylated magnetic Ni_1−x_Co_x_Fe_2_O_4_@Methionine nanoparticles, as shown in Figure 1c, peaks of the NPs were observed but the intensity of the peaks was significantly reduced due to the coating of amorphous PEG on the surface of the core-shell of the Ni_1−x_Co_x_Fe_2_O_4_@Methionine NPs; this was in good agreement with Panwar et al. [46].

As can be seen in Figure 2c, the peaks of methionine that were determined by the spot chain in the spectrum were similar to the peaks of Ni_1−x_Co_x_Fe_2_O_4_@Methionine, which clearly showed the presence of methionine on the surface of Ni_1−x_Co_x_Fe_2_O_4_ [37]. However, in magnetic Ni_1−x_Co_x_Fe_2_O_4_ NPs and methionine, van der Waals and hydrogen forces are thought to bind them. Given the amino acid groups in methionine and the presence of oxygen on the nickel structure, this possibility was reasonable.

Furthermore, before coating the Ni_1−x_Co_x_Fe_2_O_4_@Methionine NP by PEG, peaks in the regions of 3404 cm^−1^, 2835 cm^−1^, and 1058 cm^−1^ were not observed in the spectrum of the Ni_1−x_Co_x_Fe_2_O_4_@Methionine NP. These results clearly showed the surface modification of the Ni_1−x_Co_x_Fe_2_O_4_@Methionine NP with PEG. On the other hand, according to the TEM image of the Ni_1−x_Co_x_Fe_2_O_4_@Methionine NP before PEGylation, aggregation was found among the particles, which may be due to hydrogen bonding (interparticle) or van der Waals forces between the magnetic nanoparticles and methionine. After the surface coating, there was a decrease in the agglomeration, which confirmed the successful capping of Ni_1−x_Co_x_Fe_2_O_4_@Methionine with PEG.

Naproxen-loaded Ni_1−x_Co_x_Fe_2_O_4_@Methionine@PEG NPs had C=O bands that shifted from 1728 cm^−1^ to 1604 cm^−1^ because of hydrogen bonding that formed between the surface of the carrier and the naproxen [47]. Therefore, a peak in the 808 cm^−1^ region of curve e related to the bending of naproxen on the plate, resulting in the successful loading of naproxen onto the Ni_1−x_Co_x_Fe_2_O_4_@Methionine@PEG NP.

Figure 3 delineates the magnetic properties with the applied magnetic field range from 15 to 15 kOe in which the magnetization curves of the nanoparticles were measured by SQUID. The curves of the magnetization samples described a superparamagnetic function. That is important for biomaterials, making them easily trackable in the magnetic field gradient whilst maintaining the advantage of a stable and homogeneous suspension during drug delivery. When methionine was coated, bare Ni_1−x_Co_x_Fe_2_O_4_ NPs were coated and the attachment of non-magnetic groups of methionine reduced the saturation magnetization. The saturation magnetization for the Ni_1−x_Co_x_Fe_2_O_4_@Methionine@PEG NPs was also less than that for Ni_1−x_Co_x_Fe_2_O_4_@Methionine. The reduction in the magnetic properties of the Ni_1−x_Co_x_Fe_2_O_4_ NPs was due to their shells. As a result, the observed values of the saturation magnetization of the Ni_1−x_Co_x_Fe_2_O_4_ NPs with different shells confirmed the magnetic properties of these nanoparticles.

As shown in Figure 5, there was an increase in naproxen adsorption when the initial concentration of naproxen was raised. These findings showed that the adsorption of naproxen benefitted from a high loading capacity. There was a strong correlation between the loading capacity of naproxen and the initial drug concentrations; this appeared to be due to the large specific surface area of the carrier and the hydrogen bonds between the naproxen and the shells of the carriers. This was demonstrated when the loading capacity of the PEGylated Ni_1−x_Co_x_Fe_2_O_4_@Methionine NPs was raised.

As shown in Figure 6, when drugs are released into cancerous tissues, they are released into acidic intracellular lysosomes, endosomes, or diseased cells. As a result, nanoparticles could help accelerate drug release intracellularly after being internalized via endocytosis. If this is applied, anticancer drugs may have fewer side effects on healthy cells and drug losses during blood transport would be reduced. Under the same conditions, naproxen was released about 20% faster in the Ni_1−x_Co_x_Fe_2_O_4_@Methionine@PEG NPs than in the Ni_1−x_Co_x_Fe_2_O_4_@Methionine NPs because the nanoparticles could increase the intracellular drug release after being internalized by endocytosis. Under an acidic pH of 7.4, the drug release was low because a low release decreases drug loss in blood transportation; a high release at a pH of 5 facilitated the active release of anticancer drugs and the data suggested that both carriers were sensitive to the pH. The pH-dependent release was due to the strong differential in the hydrogen bonding interactions between the naproxen functional groups and the shell carrier functional groups.

The result of the MTT assay (Figure 7) showed that the cell death of naproxen loaded onto Ni_1−x_Co_x_Fe_2_O_4_@Methionine@PEG NPs was much higher than naproxen loaded onto Ni1-xCoxFe_2_O_4_@Methionine; both carriers caused more cell death than free naproxen as the method of drug absorption or the cellular absorption of the drug was not the same as that of a drug loaded onto a nanoparticle. Their cellular absorption was one of the reasons they had varying toxicities. The drug had a shorter cross-sectional effect when taken orally due to the faster release but when administered via nanoparticles, the drug had a longer effect due to the slower release and the overall effect of the drug was improved [36,37]. In high amounts, the microelement cobalt can have toxic implications despite being a cofactor of vitamin B_12_ [48]. That signifies that cobalt compounds are not particularly toxic. However, cobalt compounds can cause abnormalities, mutagenesis effects, and anemia in people [49]. Reactive oxygen species can be generated due to cobalt-induced oxidative stress [50]. In this work, the toxicity of cobalt compounds was lowered by PEGylation in various fields.

## 5. Conclusions

Recent studies have focused on magnetic formulations with amino acids or polymer shells as a drug delivery mechanism. For Ni_1−x_Co_x_Fe_2_O_4_ NPs, the methionine and PEG capping agent improved the particle stabilization, drug delivery potential in vitro characteristics, and uniform distribution of the particle size and biocompatibility. Using naproxen as a model drug at human body temperature in normal pH settings and acidic pH conditions, the Ni_1−x_Co_x_Fe_2_O_4_@Methionine@PEG NPs were shown to have drug delivery capabilities. The naproxen-loaded carrier slowly released the drug over the course of 120 h. Therefore, the Ni_1−x_Co_x_Fe_2_O_4_@Methionine@PEG nanocarrier was pH responsive, which could be a promising drug-releasing agent and facilitate the delivery of drugs to the desired tissue types in patients. The fraction cell viability was characterized by an MTT assay in which the cell death of naproxen was well-controlled through the pH-responsive magnetic nanocarriers. We demonstrated cell viability for two cancer cell lines (MCF-7 and MDA-MB-231) and a normal cell line (MCF10A), which showed that the drug-containing Ni_1−x_Co_x_Fe_2_O_4_@Methionine@PEG NPs were more potent than free naproxen and even more potent than the drug-containing Ni_1−x_Co_x_Fe_2_O_4_@Methionine NPs. There was a greater MCF-7 cell inhibition effect than in the MDA-MB-231 cell. None of the drug-loaded carriers or the free drug inhibited the growth of the normal cell line, MCF10A. The results showed significant differences in the drug-loaded Ni1-xCoxFe_2_O_4_@Methionine@PEG NP and the free drug at the same concentrations because when the drug was loaded into the carrier, which was coated by amino acids and PEG, it provided an NP with a larger surface area available for interactions, which enhanced the cell death effect and thus they imparted to the microorganisms.

## Figures and Tables

**Table 1:** The release kinetic models and the parameters obtained for the carriers.

Release Model	Equation	(a) pH = 5	(b) pH = 7.4	(c) pH = 5	(d) pH = 7.4
Zero-Order	Ct = C0 + K0t	R2 = 0.9583	R2 = 0.9205	R2 = 0.9363	R2 = 0.914
First-Order	LogC = LogC0 + Kt/2.303	R2 = 0.9842	R2 = 0.9381	R2 = 0.9607	R2 = 0.9276
Higuchi	Q = KH√*t*	R2 = 0.9900	R2 = 0.9878	R2 = 0.9794	R2 = 0.9867
Korsmeyer–Peppas	Mt/M = Ktn	R2 = 0.9852	R2 = 0.9775	R2 = 0.9775	R2 = 0.9689
		*n* = 0.3292	*n* = 0.6005	*n* = 0.4129	*n* = 0.5791

(a) Ni_1−x_Co_x_Fe_2_O_4_@Methionine NPs at pH 5, (b) Ni_1−x_Co_x_Fe_2_O_4_@Methionine NPs at pH 7.4, (c) Ni_1−x_Co_x_Fe_2_O_4_@Methionine@PEG NPs at pH 5, and (d) Ni_1−x_Co_x_Fe_2_O_4_@Methionine@PEG NPs at pH 7.4.

**Table 2:** IC_50_ values of naproxen and naproxen-loaded carriers on MCF-7 and MDA-MB-231 cells.

Cell Lines	Incubation Time	IC_50_ (μg/mL^−1^ Naproxen)	
		Free Naproxen	Naproxen Loaded onto ^a^ Naproxen Loaded onto ^b^
MCF-7	24 h	405.40 ± 97.00	117.44 ± 23.27, 138.16 ± 31.45
	48 h	352.97 ± 65.34	114.98 ± 34.26, 66.29 ± 8.71
	72 h	186.99 ± 33.83	57.45 ± 145.47, 44.03 ± 2.94
MDA-MB-231	24 h	405.06 ± 144.94	138.76 ± 26.93, 176.77 ± 34.64
	48 h	419.54 ± 295.21	98.55 ± 15.45, 92.31 ± 13.72
	72 h	218.44 ± 20.18	78.81 ± 7.71, 73.79 ± 8.46

^a^ Ni_1−x_Co_x_Fe_2_O_4_@Methionine NPs; ^b^ Ni_1−x_Co_x_Fe_2_O_4_@Methionine@PEG NPs.

## Data Availability

The data presented in this study are available on request from the corresponding author.

## References

[1] Boyle P., Levin B. (2008). World Cancer Report 2008.

[2] Akbarzadeh I., Poor A.S., Yaghmaei S., Norouzian D., Noorbazargan H., Saffar S., Cohan R.A., Bakhshandeh H. (2020). Niosomal delivery of simvastatin to MDA-MB-231 cancer cells. Drug Dev. Ind. Pharm..

[3] Akbarzadeh I., Farid M., Javidfar M., Zabet N., Shokoohian B., Arki M.K., Shpichka A., Noorbazargan H., Aghdaei H.A., Hossein-Khannazer N. (2022). The Optimized Formulation of Tamoxifen-Loaded Niosomes Efficiently Induced Apoptosis and Cell Cycle Arrest in Breast Cancer Cells. AAPS PharmSciTech.

[4] Molani S., Madadi M., Wilkes W. (2019). A partially observable Markov chain framework to estimate overdiagnosis risk in breast cancer screening: Incorporating uncertainty in patients adherence behaviors. Omega.

[5] Sarhadi M., Aryan L., Zarei M. (2020). The Estrogen Receptor and Breast Cancer: A Complete Review. Trans. Appl. Sci..

[6] Molani S., Madadi M., Williams D.L.J.M. (2020). Investigating the effectiveness of breast cancer supplemental screening considering radiologists’ bias. MedRxiv.

[7] Singh S.K., Singh S., Lillard J.W., Singh R. (2017). Drug delivery approaches for breast cancer. Int. J. Nanomed..

[8] Davahli M.R., Karwowski W., Taiar R. (2020). A System Dynamics Simulation Applied to Healthcare: A Systematic Review. Int. J. Environ. Res. Public Health.

[9] Cho H.-Y., Lee Y.-B. (2014). Nano-Sized Drug Delivery Systems for Lymphatic Delivery. J. Nanosci. Nanotechnol..

[10] Akbarzadeh I., Fatemizadeh M., Heidari F., Niri N.M. (2020). Niosomal formulation for co-administration of hydrophobic anticancer drugs into MCF-7 cancer cells. Arch. Adv. Biosci..

[11] Amale F.R., Ferdowsian S., Hajrasouliha S., Kazempoor R., Mirzaie A., Dakkali M.S., Akbarzadeh I., Meybodi S.M., Mirghafouri M. (2021). Gold nanoparticles loaded into niosomes: A novel approach for enhanced antitumor activity against human ovarian cancer. Adv. Powder Technol..

[12] Khanal P., Zargari F., Far B.F., Kumar D., Mogana R., Mahdi Y.K., Jubair N.K., Saraf S.K., Bansal P., Singh R. (2021). Integration of System Biology Tools to Investigate Huperzine A as an Anti-Alzheimer Agent. Front. Pharmacol..

[13] Shirzad M., Jamehbozorgi S., Akbarzadeh I., Aghabozorg H.R., Amini A. (2019). The Role of Polyethylene Glycol Size in Chemical Spectra, Cytotoxicity, and Release of PEGylated Nanoliposomal Cisplatin. ASSAY Drug Dev. Technol..

[14] Jamshidifar E., Yeganeh F.E., Shayan M., Yaraki M.T., Bourbour M., Moammeri A., Akbarzadeh I., Noorbazargan H., Hossein-Khannazer N. (2021). Super Magnetic Niosomal Nanocarrier as a New Approach for Treatment of Breast Cancer: A Case Study on SK-BR-3 and MDA-MB-231 Cell Lines. Int. J. Mol. Sci..

[15] Garg J., Chiu M.N., Krishnan S., Tripathi L.K., Pandit S., Far B.F., Jha N.K., Kesari K.K., Tripathi V., Pandey S. (2021). Applications of lignin nanoparticles for cancer drug delivery: An update. Mater. Lett..

[16] Roca A.G., Carmona D., Miguel-Sancho N., Bomati-Miguel O., Balas F., Piquer C., Santamaria J. (2012). Surface functionalization for tailoring the aggregation and magnetic behaviour of silica-coated iron oxide nanostructures. Nanotechnology.

[17] Yiu H.H.P. (2011). Engineering the multifunctional surface on magnetic nanoparticles for targeted biomedical applications: A chemical approach. Nanomedicine.

[18] Cheong S., Ferguson P., Feindel K.W., Hermans I.F., Callaghan P.T., Meyer C., Slocombe A., Su C.-H., Cheng F.-Y., Yeh C.-S. (2011). Simple Synthesis and Functionalization of Iron Nanoparticles for Magnetic Resonance Imaging. Angew. Chem. Int. Ed..

[19] Cho N.-H., Cheong T.-C., Min J.H., Wu J.H., Lee S.J., Kim D., Yang J.-S., Kim S., Kim Y.K., Seong S.-Y. (2011). A multifunctional core–shell nanoparticle for dendritic cell-based cancer immunotherapy. Nat. Nanotechnol..

[20] He X., Gao J., Gambhir S.S., Cheng Z. (2010). Near-infrared fluorescent nanoprobes for cancer molecular imaging: Status and challenges. Trends Mol. Med..

[21] Amiri S., Shokrollahi H. (2013). The role of cobalt ferrite magnetic nanoparticles in medical science. Mater. Sci. Eng. C.

[22] Comes R., Liu H., Khokhlov M., Kasica R., Lu J., Wolf S.A. (2012). Directed Self-Assembly of Epitaxial CoFe_2_O_4_–BiFeO_3_ Multiferroic Nanocomposites. Nano Lett..

[23] Boran G., Tavakoli S., Dierking I., Kamali A.R., Ege D. (2020). Synergistic effect of graphene oxide and zoledronic acid for osteoporosis and cancer treatment. Sci. Rep..

[24] Far B.F., Asadi S., Naimi-Jamal M.R., Abdelbasset W.K., Shahrivar A.A. (2021). Insights into the interaction of azinphos-methyl with bovine serum albumin: Experimental and molecular docking studies. J. Biomol. Struct. Dyn..

[25] Sivakumar M., Kanagesan S., Babu R.S., Jesurani S., Velmurugan R., Thirupathi C., Kalaivani T. (2012). Synthesis of CoFe_2_O_4_ powder via PVA assisted sol–gel process. J. Mater. Sci. Mater. Electron..

[26] Foroughi F., Hassanzadeh-Tabrizi S., Bigham A. (2016). In situ microemulsion synthesis of hydroxyapatite-MgFe_2_O_4_ nanocomposite as a magnetic drug delivery system. Mater. Sci. Eng. C.

[27] Joshi H.M., Lin Y.P., Aslam M., Prasad P.V., Schultz-Sikma E.A., Edelman R., Meade T., Dravid V.P. (2009). Effects of Shape and Size of Cobalt Ferrite Nanostructures on Their MRI Contrast and Thermal Activation. J. Phys. Chem. C.

[28] Verde E.L., Landi G., Gomes J., Sousa M.H., Bakuzis A.F. (2012). Magnetic hyperthermia investigation of cobalt ferrite nanoparticles: Comparison between experiment, linear response theory, and dynamic hysteresis simulations. J. Appl. Phys..

[29] Fan H., Li B., Shi Z., Zhao L., Wang K., Qiu D. (2018). A fibrous morphology silica-CoFe_2_O_4_ nanocarrier for anticancer drug delivery. Ceram. Int..

[30] Sun C., Lee J.S.H., Zhang M. (2008). Magnetic nanoparticles in MR imaging and drug delivery. Adv. Drug Deliv. Rev..

[31] Grange S., Chettiar K., Arestu M., Pilarski P., Smitham P., Loizidou M., Jell G. (2013). Nanotechnology and medical devices: Risk, regulation and ‘meta’ registration. World J. Eng..

[32] Ghorbani M., Hamishehkar H., Arsalani N., Entezami A.A. (2015). Preparation of thermo and pH-responsive polymer@ Au/Fe_3_O_4_ core/shell nanoparticles as a carrier for delivery of anticancer agent. J. Nanopart. Res..

[33] Unsoy G., Khodadust R., Yalcin S., Mutlu P., Gunduz U. (2014). Synthesis of Doxorubicin loaded magnetic chitosan nanoparticles for pH responsive targeted drug delivery. Eur. J. Pharm. Sci..

[34] Sundaresan V., Menon J., Rahimi M., Nguyen K.T., Wadajkar A.S. (2014). Dual-responsive polymer-coated iron oxide nanoparticles for drug delivery and imaging applications. Int. J. Pharm..

[35] Fan H., Li L., Zhou S., Liu Y.-Z. (2016). Continuous preparation of Fe_3_O_4_ nanoparticles combined with surface modification by L-cysteine and their application in heavy metal adsorption. Ceram. Int..

[36] Wang Y., Xiao Y., Gao G., Chen J., Hou R., Wang Q., Liu L., Fu J. (2017). Conductive graphene oxide hydrogels reduced and bridged by l-cysteine to support cell adhesion and growth. J. Mater. Chem. B.

[37] Eshrati Yeganeh F., Yousefi M., Hekmati M., Bikhof M. (2021). An experimental research on pH-responsive amino acid-coated Ni_(1−x)_ Co_x_Fe_2_O_4_ nanoparticles as a nanocarrier for drug delivery and biological applications. Chem. Pap..

[38] Zarghi A., Arfaei S. (2011). Selective COX-2 inhibitors: A review of their structure-activity relationships. Iran. J. Pharm. Res..

[39] Akbarzadeh I., Tabarzad M., Khazraei H., Ostovan V. (2021). Development of a novel niosomal formulation for Gabapentin. Iran. J. Colorectal Res..

[40] Sahrayi H., Hosseini E., Karimifard S., Khayam N., Meybodi S.M., Amiri S., Bourbour M., Far B.F., Akbarzadeh I., Bhia M. (2021). Co-Delivery of Letrozole and Cyclophosphamide via Folic Acid-Decorated Nanoniosomes for Breast Cancer Therapy: Synergic Effect, Augmentation of Cytotoxicity, and Apoptosis Gene Expression. Pharmaceuticals.

[41] Vajedi F.S., Dehghani H., Zarrabi A. (2021). Design and characterization of a novel pH-sensitive biocompatible and multifunctional nanocarrier for in vitro paclitaxel release. Mater. Sci. Eng. C.

[42] Lingaraju K., Basavaraj R., Jayanna K., Bhavana S., Devaraja S., Swamy H.K., Nagaraju G., Nagabhushana H., Naika H.R. (2021). Biocompatible fabrication of TiO_2_ nanoparticles: Antimicrobial, anticoagulant, antiplatelet, direct hemolytic and cytotoxicity properties. Inorg. Chem. Commun..

[43] Gong P., Wang Y., Zhang P., Yang Z., Deng W., Sun Z., Yang M., Li X., Ma G., Deng G. (2020). Immunocyte Membrane-Coated Nanoparticles for Cancer Immunotherapy. Cancers.

[44] Li H., Zhou F., Li L., Zheng Y. (2016). Design and development of novel MRI compatible zirconium-ruthenium alloys with ultralow magnetic susceptibility. Sci. Rep..

[45] Yalcin B., Ozcelik S., Icin K., Senturk K., Arda L. (2021). Structural, optical, magnetic, photocatalytic activity and related biological effects of CoFe_2_O_4_ ferrite nanoparticles. J. Mater. Sci. Mater. Electron..

[46] Panwar V., Kumar P., Bansal A., Ray S.S., Jain S.L. (2015). PEGylated magnetic nanoparticles (PEG@Fe_3_O_4_) as cost effective alternative for oxidative cyanation of tertiary amines via CH activation. Appl. Catal. A Gen..

[47] Patnaika S., Rao K.M., Sai V. (2017). Cytotoxicity Studies on Naproxen and Piroxicam Nanoformulations. J. Nanopart. Res..

[48] Bondarenko O.M., Ivask A., Kahru A., Vija H., Titma T., Visnapuu M., Joost U., Pudova K., Adamberg S., Visnapuu T. (2016). Bacterial polysaccharide levan as stabilizing, non-toxic and functional coating material for microelement-nanoparticles. Carbohydr. Polym..

[49] Dolara P. (2014). Occurrence, exposure, effects, recommended intake and possible dietary use of selected trace compounds (aluminium, bismuth, cobalt, gold, lithium, nickel, silver). Int. J. Food Sci. Nutr..

[50] Gari M.K., Lemke P., Lu K.H., Laudadio E.D., Henke A.H., Green C.M., Pho T., Hoang K.N.L., Murphy C.J., Hamers R.J. (2021). Dynamic aqueous transformations of lithium cobalt oxide nanoparticle induce distinct oxidative stress responses of *B. subtilis*. Environ. Sci. Nano.

